# Location-independent feature binding in visual working memory for sequentially presented objects

**DOI:** 10.3758/s13414-021-02245-w

**Published:** 2021-04-16

**Authors:** Sebastian Schneegans, William J. Harrison, Paul M. Bays

**Affiliations:** 1 Department of Psychology, University of Cambridge, Downing Street, Cambridge, CB2 3EB UK; 2 Present Address: Queensland Brain Institute, University of Queensland, St Lucia, Queensland 4072 Australia

**Keywords:** Visual working memory, Feature binding, Cued recall, Perceptual interference

## Abstract

Spatial location is believed to have a privileged role in binding features held in visual working memory. Supporting this view, Pertzov and Husain (*Attention, Perception, & Psychophysics*, *76*(7), 1914–1924, 2014) reported that recall of bindings between visual features was selectively impaired when items were presented sequentially at the same location compared to sequentially at different locations. We replicated their experiment, but additionally tested whether the observed impairment could be explained by perceptual interference during encoding. Participants viewed four oriented bars in highly discriminable colors presented sequentially either at the same or different locations, and after a brief delay were cued with one color to reproduce the associated orientation. When we used the same timing as the original study, we reproduced its key finding of impaired binding memory in the same-location condition. Critically, however, this effect was significantly modulated by the duration of the inter-stimulus interval, and disappeared if memoranda were presented with longer delays between them. In a second experiment, we tested whether the effect generalized to other visual features, namely reporting of colors cued by stimulus shape. While we found performance deficits in the same-location condition, these did not selectively affect binding memory. We argue that the observed effects are best explained by encoding interference, and that memory for feature binding is not necessarily impaired when memoranda share the same location.

**Supplementary Information:**

The online version contains supplementary material available at (10.3758/s13414-021-02245-w).

## Introduction

To accurately recall details of a visual scene, we need to have encoded not only the individual features that were present, but also the specific conjunctions of features that constitute different objects (Treisman, 1996). In change detection tasks, limitations in memory for feature conjunctions are assessed by comparing performance between test displays that involve recombinations of sample features versus substitution with novel features (Wheeler & Treisman, 2002). In delayed reproduction tasks, failures to accurately memorize or retrieve feature bindings are reflected in “swap” errors, in which participants report the feature of an item that is not the cued target (Bays, Catalao, & Husain, 2009).

There is substantial evidence that object location plays a special role in visual working memory (VWM), and in feature binding in particular. Convergent findings from behavioral and imaging studies show that object locations—unlike other visual features—are encoded and maintained in working memory automatically, even when not task-relevant (Chen & Wyble, 2015; Elsley & Parmentier, 2015; Foster, Bsales, Jaffe, & Awh, 2017; Cai, Sheldon, Yu, & Postle, 2019). Change detection performance tends to be improved if stimulus locations remain fixed between the sample and test array (Hollingworth, 2007), and location is a particularly effective cue in delayed reproduction tasks (Rajsic, Swan, Wilson, & Pratt, 2017). Moreover, cuing an item held in working memory draws spatial attention and biases eye movements towards the location where the item was seen, even if neither the cue nor the feature to be reported are spatial (Griffin & Nobre, 2003; Theeuwes, Kramer, & Irwin, 2011; van Ede, Chekroud, & Nobre, 2019).

The latter finding is consistent with the idea that location also plays an important role in binding non-spatial features of an object in working memory. This view was popularized by the influential study of Treisman and Zhang (2006), based on specific effects that task-irrelevant location changes had on response behavior in different change detection tasks. More recently, the mechanisms of feature binding have been investigated using delayed reproduction tasks in which multiple features of a cued item have to be reported. The patterns of error correlations in these tasks indicate that features like color and orientation are bound independently to an item’s location in a stimulus array (Bays, Wu, & Husain, 2011; Fougnie & Alvarez, 2011), and are bound to each other only indirectly via this shared location (Schneegans & Bays, 2017; Kovacs & Harris, 2019).

Importantly, distinct visual objects may be separated in time as well as, or instead of, space. The temporal order of sequentially presented stimuli can be recalled with high accuracy (van Asselen, Van der Lubbe, & Postma, 2006), and ordinal position can be used reliably as a cue to indicate the target item (Harrison & Tong, 2009). A study by (Gorgoraptis, Catalao, Bays, & Husain, 2011) found that participants in a delayed reproduction task could retrieve the binding between stimulus colors and orientations with performance far above chance level when items were presented sequentially, either at different locations or all at the same location. The latter condition demonstrates that memory for feature binding cannot be mediated exclusively by spatial location. However, the same study also observed that the proportions of swap errors and random responses were substantially higher in both sequential presentation conditions compared to simultaneous presentation of a sample array (see also Allen, Baddeley, & Hitch, 2006, for related findings in change detection).

A subsequent study by Pertzov and Husain (2014) directly investigated whether memory for feature binding was impaired when sample stimuli shared the same location. In a delayed reproduction task, they sequentially presented four colored, oriented bars in each trial, and then cued participants with the color of one bar to report its orientation. In separate blocks of trials, the bars within a trial either appeared all at the same location, or each appeared at a different location. Even though object location was not relevant for the task, the study found a specific increase in the proportion of swap errors when items were presented at the same location. The authors surmised that when features of multiple objects are bound to the same spatial location, they are more likely to be confused at recall. This result suggests that binding via location, while not the sole mechanism for feature binding in VWM, may still be the dominant or preferred mechanism, and that alternative ways of memorizing feature conjunctions may be less reliable.

However, Harrison and Bays (2018) found discrepant results in a study aimed at investigating crowding effects (Pelli & Tillman, 2008) in VWM, even though the task they used was very similar to that of Pertzov & Husain. Participants had to report the orientation of a bar cued by its color, and the locations of the sequentially presented sample stimuli were varied to be either closely spaced (within the range where crowding effects would be observed in perceptual tasks) or further apart. The study found evidence against an effect of spatial proximity on recall performance for sequentially presented items.

Several small differences in the study designs could be responsible for the conflicting results. First, the stimuli in the crowded condition of Harrison & Bays did not precisely share the same location. However, they were close enough to each other to cause perceptual interference when stimuli were presented simultaneously, and it should have been hard for participants to even detect the difference in locations when stimuli were presented sequentially. Second, participants in the crowding study were presented with only three stimuli per trial, instead of four in the experiment of Pertzov & Husain. But even with three stimuli a moderate number of swap errors occurred, the frequency of which should have been modulated by the task condition if location was critical for binding.

A final difference between the two studies was in the *temporal* proximity between sequentially presented sample items—i.e., the presentation time for each item and the inter-stimulus interval (ISI). Pertzov and Husain (2014) presented items quite rapidly (200 ms sample and 300-ms ISI), while Harrison and Bays (2018) allowed twice as much time for each item (500 ms sample and 500 ms ISI). A recent study by Ahmad et al., (2017) observed decreased recall precision and a higher proportions of swap errors in VWM task when stimuli were presented close to each other both in space and in time, but the effect of spatial proximity disappeared with a longer ISI (500 ms). While this experiment did not require binding between different non-spatial features, it suggests that interference or competition between rapidly presented stimuli can impair subsequent recall performance.

The aim of the present study is to resolve these conflicting findings, and to determine whether working memory for feature binding is necessarily impaired when memoranda share the same location, even when they are well separated in time. To this end, we reproduced the study of Pertzov and Husain (2014) with stimulus timing as an additional factor in a within-subject design. To preview our results, we replicated the effect of shared location on swap errors observed in the original study when using the same stimulus timing, but observed no effect when stimuli were presented with longer ISIs. In a second experiment, we tested whether the effects observed in this first experiment generalize to other feature combinations, using colored shapes as stimuli. We found evidence for impaired performance when items were presented rapidly at the same location, but the impairment could not be attributed to a selective increase in the proportion of swap errors.

## Experiment 1

### Methods

#### Stimuli and procedure

The study used a 2 (location condition) × 2 (ISI condition) within-subject design, with conditions blocked. Stimuli and procedure closely followed Experiment 1 of Pertzov and Husain (2014), with the exception of the added ISI condition and a small change in the cue stimulus described below.

Twelve participants (nine female, mean age 26.5 years) performed the experiment after giving informed consent in accordance with the Declaration of Helsinki. All reported normal or corrected-to-normal visual acuity and showed normal color vision in an Isihara color test. The number of participants was determined by a Bayesian stopping criterion (see “Statistical analysis”). Participants were seated in front of a computer monitor (27” LCD screen with a refresh rate of 166 Hz) at a viewing distance of 60 cm, with their head position stabilized by a head rest. Gaze direction was monitored using an infrared eye tracker (EyeLink 1000, SR Research) operating at 1000 Hz.

**Fig. 1:**
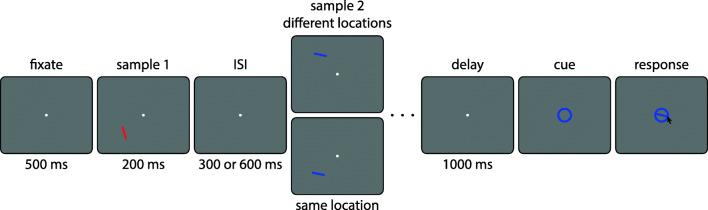
Task design in Experiment 1. Four colored oriented bars were presented sequentially in each trial (only the first two are shown here), either at the same location or all at different locations. After a memory delay, the participant had to report the orientation of one bar cued by its color

The task design is illustrated in Fig. 1. Each trial began with the presentation of a central fixation point, a white disk with a diameter of 0.25 degree of visual angle (dva), shown on a medium gray background. After 500 ms of maintained fixation on this point, four colored, oriented bars were presented sequentially in the periphery. Each bar had a length of 2 dva and a width of 0.3 dva, a unique color (red, green, blue, or yellow, in random order within each trial) and a random orientation drawn with uniform probability from the range of possible bar orientations [0^∘^,180^∘^), with the constraint that the orientations of any two bars had to differ by at least 10^∘^. In the *different location* condition, each bar was presented in a random location on an invisible circle with a radius of 6 dva around the fixation point, with a minimum distance of 3 dva between the centers of any two bars. In the *same location* condition, all bars within a trial were presented in the same location on this circle, but the location still varied randomly from trial to trial. Each bar was presented for 200 ms, with an ISI of 300 ms in the *short ISI* condition, and 600 ms in the *long ISI* condition.

After a memory delay of 1000 ms following the last sample stimulus, the fixation point was replaced by a central color cue in the form of an annulus with an inner diameter matching the length of the oriented bars. Participants then had to report the memorized orientation of the target stimulus (the bar matching this cue color) with a mouse. A probe bar appeared when the mouse pointer was first moved over the annulus, and its orientation could be continuously adjusted (following the angular position of the mouse pointer). The response was finalized by a mouse click. We used a colored annulus as the cue stimulus instead of the randomly oriented colored bar employed by Pertzov and Husain (2014) to minimize any possible interference of the cue with orientation memory (Souza, Rerko, & Oberauer, 2016). If participants lost fixation before onset of the response cue, the trial was aborted and repeated later in the same block.

For each of the four combinations of location (same or different) and ISI (short or long) conditions, participants completed 120 trials divided into three consecutive blocks of 40 trials each. Within each block, the sample item at each of the four ordinal positions was tested ten times (randomly interleaved). The order of conditions was counterbalanced across participants. Stimulus presentation and response collection were controlled using MATLAB (The MathWorks, Inc.) with the Psychophysics Toolbox (Brainard, 1997; Pelli, 1997; Kleiner, Brainard, & Pelli, 2007) and Eyelink Toolbox (Cornelissen, Peters, & Palmer, 2002) extensions.

#### Response distributions and mixture model fits

We obtained histograms of response errors (angular deviations between the reported orientation and the orientation of the target item in each trial) for each participant and each task condition to visualize response distributions. We also determined histograms of response deviations from the orientations of the non-target items in each trial. A central peak in these histograms signifies the presence of swap errors (i.e., erroneous report of a non-target’s orientation). However, the minimum separation between the orientations of different items within a trial causes the histograms of non-target deviations expected by chance (i.e., without any swap errors) to be non-uniform.

To remove this effect, we applied a correction to these histogram using a shuffling method (Schneegans & Bays 2017; code available at https://bayslab.com/toolbox). We determined the deviations of the non-target orientations from the target orientation in one trial, A, and added these to the target orientation in another trial, B. This yields a new set of non-target orientations for trial B that still obey the minimum distance requirements both to the target feature and among each other, but are unrelated to the response in this trial. We then determined the deviation of the response made in trial B from these shuffled non-target orientations. We did this for every possible pair of trials (separately for each participant and task condition) to obtain an expected histogram of response deviations from non-target features in the absence of swap errors. Finally, we subtracted this expected histogram from the original histogram to determine the corrected histogram. Any remaining central peak in the corrected histogram indicates the occurrence of swap errors.

Response distributions were fit with a three-component mixture model (Bays et al., 2009). In this descriptive model, each response is assumed to be drawn either from a von Mises (circular normal) distribution around the target value, a von Mises distribution centered on the feature of one non-target item in the same trial (a swap error), or from a uniform distribution. This yields the following probability density function,

1 $$ p(\hat{\theta}) = p_{T} \phi_{\circ}(\hat{\theta}; \theta, \kappa) + p_{NT} \frac{1}{3} \sum\limits_{i = 1}^{3} \phi_{\circ}(\hat{\theta}; \varphi_{i}, \kappa) + p_{U} \frac{1}{2\pi}. $$

Here, $\hat {\theta }$ is the reported value, *𝜃* is the true target value, and *φ*_*i*_ are the feature values of the non-targets in the trial. We denote with $\phi _{\circ }(\hat {\theta }; \mu , \kappa )$ the von Mises distribution centered at *μ* with concentration *κ*, evaluated at the value $\hat {\theta }$. The model has three free parameters, namely the proportions of swap errors, *p*_*NT*_, and of uniform responses, *p*_*U*_ (with the proportion of target responses *p*_*T*_ = 1 − *p*_*NT*_ − *p*_*U*_), and the concentration parameter *κ* of the von Mises distribution.

A separate maximum-likelihood fit of the model was obtained for the response distribution of each participant in each of the four experimental conditions (using code available at https://bayslab.com/toolbox). We note that for the orientation responses, all feature values (which were in the range [0^∘^,180^∘^)) were scaled by a factor of two before applying the mixture model so that the von Mises distribution could be used in its standard formulation over the whole circle.

Following Pertzov and Husain (2014), we also used a simple heuristic to estimate the number of target responses and swap errors independently from the model fit. We determined the proportion $\tilde {p}_{T}$ of responses that fell within a certain range of the target feature (15^∘^), as well as the proportion $\tilde {p}_{NT}$ that fell within the same range around any of the non-target features in a trial. This measure does not make any specific assumptions about the shape of response distributions, and only relies on the expectation that an increase in the proportion of target or non-target responses should produce an increase in the frequency of response values in the vicinity of the target or non-target feature values, respectively. Note that for estimating the proportion of non-target responses, we use the histograms without correction for minimum feature separation. While the correction is useful for visualizing the occurrence of swap errors, it does not provide any specific advantages when comparing response frequencies across conditions. Using the uncorrected histograms reduces the reliance on any prior assumptions about response distributions, and also directly matches the method of Pertzov and Husain (2014).

Finally, we assessed the occurrence of swap errors at different temporal or spatial distances between target and non-target items, adapting a method used in Schneegans and Bays (2017). For the temporal distance effect, we grouped all non-target items according to their ordinal position relative to the target item (from preceding the target by three steps to succeeding it by three steps). For each group, we then determined the mean absolute deviation (MAD) of the response in a trial from the non-target feature values in the same trial. If the MAD is below the level expected by chance (in the absence of swap errors), this indicates the occurrence of swap errors specifically for items at a certain temporal separation.

For assessing effects of spatial distance, we similarly grouped non-targets according to their angular distance from the target location (in the different-location conditions only). We used four distance bins, the first covering angular distances up to 67.5^∘^, and each other spanning a 37.5^∘^ range up to 180^∘^ (the minimum spatial distance of 3 dva used in the experiment translates to an angular distance of approximately 30^∘^, so this spacing produces nearly equal numbers of non-targets falling into each bin). We then again determined the MAD of the response in each trial from the non-targets in the same trial that fall within a specific distance bin. The minimum distance between items’ feature values within a trial also affects the expected MAD in the absence of swap errors, which would otherwise be 45^∘^. We determined the expected deviation using the same shuffling method as described above, by determining the MAD of a response value from all shuffled non-target feature values.

#### Statistical analysis

We used Bayesian statistics to determine the evidence for an effect of the different experimental conditions on recall performance. We applied a two-factors (location condition and ISI condition) repeated measures Bayesian ANOVA on the obtained mixture model parameters as well as the heuristic measures for the proportion of target responses and swap errors. Subsequent paired-sample Bayesian *t* tests were performed where the ANOVA revealed evidence for interaction effects. We additionally performed a three-factor repeated measures Bayesian ANOVA on the mean absolute response errors, with ordinal position of the cued item as an additional factor, and on the MADs of responses from non-target features, with temporal separation as third factor. For the effects of spatial distance between targets and non-targets, we performed an ANOVA with factors ISI and distance bin (since this measure is only applicable for the different-location conditions). All tests were performed in JASP (version 0.14.0.0) using the standard parameters. For ANOVAs, we report the evidence in favor of inclusion of each factor and interaction, BF_incl_, estimated across matched models. For Bayesian *t* tests, we report the evidence in favor of an effect over the null hypothesis, BF_10_.

We further employed a Bayesian stopping criterion (Rouder, 2014) to determine the number of participants in the experiment. The main hypothesis tested in Experiment 1 was that the effect of the location condition on the proportion of swap errors observed by (Pertzov & Husain, 2014) is modulated by the length of the ISI. This predicts an interaction effect that can be tested in the Bayesian ANOVA; however, this cannot be computed analytically in standard Bayesian methods and is instead estimated by sampling, making it less suitable for a stopping criterion. We therefore used the difference-of-differences in the proportion of swap errors between conditions, *Δp*_*NT*_, as a proxy for the interaction effect:

2 $$ \begin{array}{@{}rcl@{}} {\varDelta} p_{NT} = \left( p_{NT}(\mathrm{different, short}) - p_{NT}(\mathrm{same, short}) \right) \notag\\ - \left( p_{NT}(\mathrm{different, long}) - p_{NT}(\mathrm{same, long}) \right) \end{array} $$

We used a one-sample Bayesian *t* test as basis for a stopping criterion in the number of participants, terminating the experiment after strong evidence (Bayes factor > 10) either in favor or against the hypothesis that *Δp*_*NT*_≠ 0 was found, or after a maximum of 20 participants when this criterion was not reached. This Bayesian *t* test constitutes a more conservative criterion for stopping than the evidence for an interaction effect in the ANOVA.

### Results

In Experiment 1, we sequentially presented four colored, oriented bars, and participants had to report the orientation of one bar cued by its color. Two factors were varied in a blocked within-subjects design: stimulus location (same or different for the stimuli within a trial) and ISI (300 or 600 ms).

**Fig. 2:**
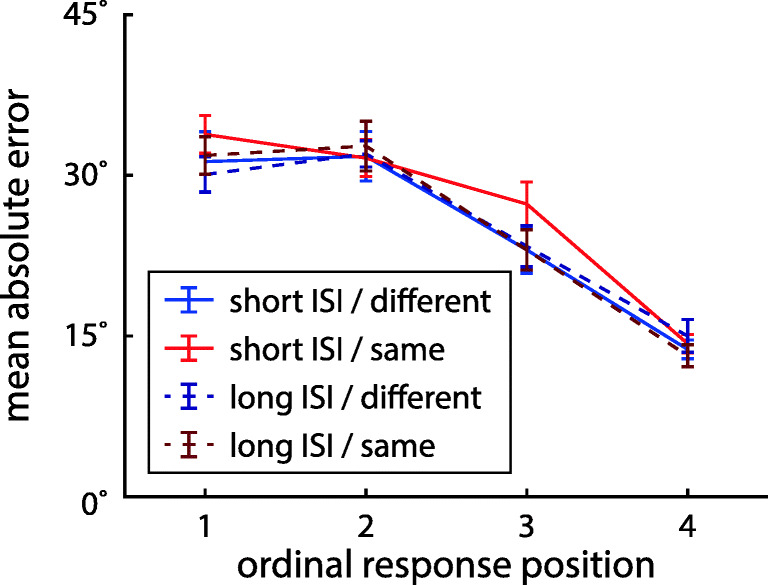
Mean absolute error in reported orientation for target items at different ordinal positions in each task condition of Experiment 1. *Error bars* indicate ± 1 standard error (SE)

We first determined the effects of the task conditions and the ordinal position of the cued item on mean absolute response error, as a model-free measure of performance (Fig. 2). A three-factor Bayesian ANOVA (with factors location, ISI, and ordinal position) produced overwhelming evidence for an effect of ordinal position (BF_incl_ = 2.72 ⋅ 10^45^). There was weak evidence against an effect of location (BF_incl_ = 0.37) and moderate evidence against an effect of ISI (BF_incl_ = 0.25), as well as weak to moderate evidence against any interaction effects (all BF_incl_ between 0.14 and 0.42). This suggests that overall recall performance was comparable across task conditions. The effect of ordinal position takes the form of a recency benefit, which is broadly consistent with previous studies (Gorgoraptis et al., 2011).

**Fig. 3:**
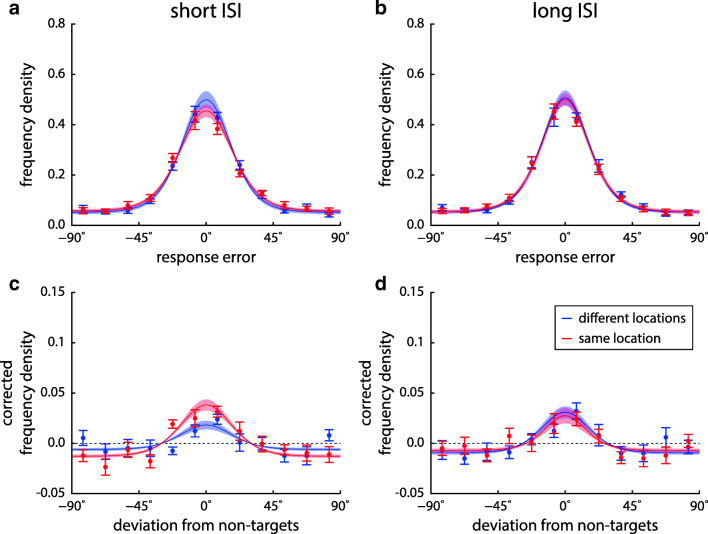
Response distributions relative to target (**a** & **b**) and non-target feature values (**c** & **d**) in Experiment 1, with corresponding mixture model fits. Histograms are shown as data points with error bars, and model fits as *solid lines with shaded areas*, both indicating ± 1 SE. Different location conditions are color coded (*blue* for different location, *red* for same location), while histograms for different ISI conditions are shown in separate panels (**a** & **c** for short ISI, **b** & **d** for long ISI). Histograms of deviations from non-target features are corrected for effects of minimum feature distance between sample items within a trial, in such a way that the distributions would be uniform at zero if non-targets had no effects on responses. Note that model fits are based on single-trial data rather than the binned data shown in the plots

**Fig. 4:**
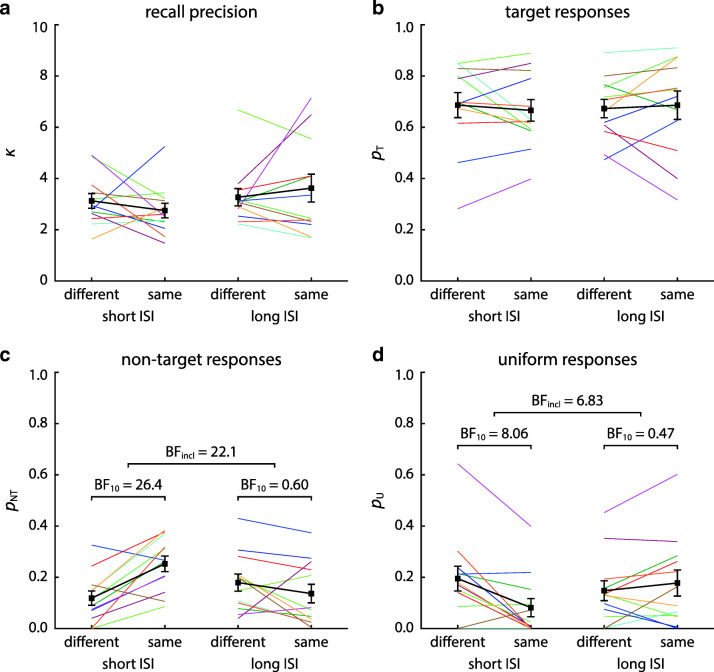
Parameter estimates of mixture model fits for Experiment 1. Separate panels show estimates of the concentration parameter *κ* (**a**), proportion of target responses *p*_*T*_ (**b**), proportion of swap errors *p*_*NT*_ (**c**), and proportion of uniform responses *p*_*U*_ (**d**) for the four experimental conditions. *Colored lines* show estimates for individual participants, while *thicker black lines* show the average across participants, with error bars indicating ± 1 SE. Concentration parameters reflect precision after scaling the orientation data up to the range [− 180^∘^,180^∘^), in order to use the standard formulation of the von Mises distribution

To analyze effects of task conditions on specific response errors, we fit a mixture model (Bays et al., 2009) to the response distributions of each participant in each condition (pooled over ordinal positions). This yields estimates of recall precision and proportions of target, non-target, and uniform responses. Histograms and model fits of response deviations from target and non-target orientations are shown in Fig. 3, and estimated mixture model parameters in Fig. 4.

Based on the previous findings of Pertzov and Husain (2014), we expected to find a specific effect of location on the proportion of swap errors for short ISIs (with more swap errors in the same-location condition). Based on the results of Harrison and Bays (2018), however, we hypothesized that this effect would not generalize to long ISIs, and that we consequently would find an interaction effect of location and ISI conditions on the proportion of swap errors. We employed a Bayesian stopping criterion for this interaction effect (expressed as a difference of differences) to determine the number of participants in the experiment. The criterion was reached after 12 participants, with strong evidence in favor of an interaction (BF_10_ = 15.5).

A subsequent Bayesian ANOVA confirmed this interaction effect (BF_incl_ = 22.1), while results were inconclusive regarding a single-factor effect of location (BF_incl_ = 0.76) and showed weak evidence against an effect of ISI (BF_incl_ = 0.41). Separate Bayesian *t* tests on the effect of location within each ISI condition confirmed that the interaction took the form that we had hypothesized (Fig. 4c): For short ISIs, there was strong evidence that the proportion of swap errors was higher in the same-location than in the different-location condition (BF_10_ = 26.4), while for long ISIs, there was weak evidence against an effect of location (BF_10_ = 0.60).

The occurrence of swap errors can be visualized by plotting the histograms of response deviations from the non-targets of each trial, as shown in Fig. 3c and d (corrected by subtracting the distribution that would be expected in the absence of swap errors). Following the method of Pertzov and Husain (2014), we determined the proportion of trials in the two central bins of this histogram (within ± 15^∘^ of the non-target feature) as a heuristic measure for the proportion of swap errors, and compared them across conditions. However, a Bayesian ANOVA on this measure was inconclusive regarding an interaction of location and ISI (BF_incl_ = 1.21), even though within each ISI condition, the findings from the mixture model were supported (higher proportion of swap errors for the same-location condition with short ISI, BF_10_ = 18.8, no effect of location for long ISI, BF_10_ = 0.29). Visual inspection of the histograms suggests that many trials outside of the range of ± 15^∘^ contributed to the proportion of swap errors, and a post hoc test indeed showed moderate evidence for an interaction effect when the range was extended to ± 30^∘^ (BF_incl_ = 6.03).

We also applied the Bayesian ANOVA to the other parameters of the mixture model fit. We note that comparisons for these parameters are more likely to show weak or inconclusive evidence since our sample size was determined by a stopping rule on the proportion of swap errors, being the main variable of interest and the one we expected to show the largest effects.

For the concentration parameter *κ* (Fig. 4a), the results provided weak to moderate evidence against an effect of location (BF_incl_ = 0.29), ISI (BF_incl_ = 0.82), and an interaction of these factors (BF_incl_ = 0.61). Similarly, we found weak-to-moderate evidence against an effect of location (BF_incl_ = 0.29), ISI (BF_incl_ = 0.29), and an interaction (BF_incl_ = 0.41) on the proportion of target responses (Fig. 4b). Applying the heuristic approach to estimate the proportion of target responses from the response histograms likewise yielded weak evidence against an effect of location (BF_incl_ = 0.52) or ISI (BF_incl_ = 0.42), and results were equivocal regarding an interaction effect (BF_10_ = 1.30).

For the proportion of uniform responses (Fig. 4c), there was weak evidence against an effect of location (BF_incl_ = 0.72) and ISI (BF_incl_ = 0.39). However, we found moderate evidence for an interaction of these two factors (BF_incl_ = 6.83). Subsequent Bayesian *t* test showed that the form of this interaction was complementary to the one observed for the proportion of swap errors: At short ISIs, the proportion of responses captured by the uniform component of the model was lower in the same-location condition compared to the different-location condition (BF_10_ = 8.06), while for long ISIs, there was weak evidence against an effect of location (BF_10_ = 0.47).

**Fig. 5:**
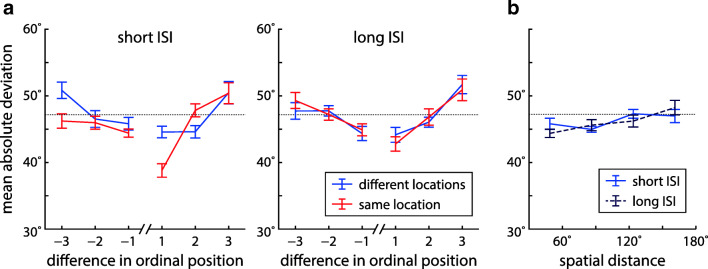
Effects of temporal and spatial proximity of non-targets on orientation responses in Experiment 1. All plots show the MADs of response values from non-target features in the same trial, grouped by temporal or spatial distance between the non-target item and the target. The *dotted line* indicates the expected MAD in the absence of swap errors and bias effects (averaged across participants and conditions). **a** MADs for non-targets with different temporal distances to the target, for all combinations of ISI and location condition. *Negative values* for the difference in ordinal position indicate non-targets that precede the target, *positive values* indicate non-targets that follow the target. **b** MADs for non-targets with different spatial distances (binned, measured as difference in angular location) in the different-location condition for short and long ISIs

To further elucidate the patterns of swap errors in different task conditions, we analyzed the deviation of responses from non-target features at different temporal separations (based on the ordinal positions of target and non-target items in the sequence of stimuli within each trial) and for different spatial distances (based on angular locations). Effects of temporal separation are shown in Fig. 5a. If swap errors occur for certain separations, this will decrease the MAD below chance levels (shown as dotted line; Schneegans & Bays 2017). Due to the minimum distance between the features of different items within a trial, the MAD for other separations can then be increased above the chance level.

A repeated measures Bayesian ANOVA with factors location, ISI, and temporal separation produced overwhelming evidence for an effect of temporal separation (BF_incl_ = 2.98 ⋅ 10^18^), with MAD values below chance level for non-targets immediately preceding or succeeding the target item (Table S1). We also found weak evidence for a location-separation interaction (BF_incl_ = 1.58) and a location-ISI-separation interaction (BF_incl_ = 1.73). Notably, the MAD for the item immediately following the target is decreased in the same-location condition for short ISIs, in which we observed a specific increase in swap errors. All other factors and interactions showed evidence against an effect (BF_incl_ between 0.04 and 0.69).

We also assessed the effects of spatial distance in the different location conditions (Fig 5b). An ANOVA with factors ISI and distance bin showed strong evidence for an effect of target-to-non-target distance (BF_incl_ = 16.9), with lower MADs in the two bins for smaller distances (Table S2). We found evidence against an effect of ISI (BF_incl_ = 0.22) and an interaction (BF_incl_ = 0.49).

### Discussion

We successfully reproduced a key finding from the main experiment of Pertzov and Husain (2014), namely that presenting memory sample stimuli sequentially at the same location selectively increased the proportion of swap errors when using short ISIs. However, we also found strong evidence for an interaction of this effect with ISI, and no positive evidence for an effect of location remained at longer ISIs. This confirms our main hypothesis.

Pertzov and Husain (2014) had tested the effect of location at longer ISIs in a control experiment (reported in their supplementary material) and found support for the same effect as for short ISIs. Converting the result of their *t* test (t(7) = 2.6, *p* = 0.03) into a Bayes factor shows that their evidence for a location effect is only weak (BF_10_ = 2.46), while we found weak evidence *against* such an effect (BF_10_ = 0.60). It therefore remains an open question whether or not some location effect persists at the longer ISI. However, the within-subjects design employed here produced clear evidence that the effect decreases with increasing ISI.

We note that even in the short ISI condition, presenting all sample items at the same location did not lead to a complete breakdown of color-orientation binding. Although the estimated proportion of swap errors approximately doubled compared to the different-location condition (from 12% to 25%), a majority of responses was still classified as target reports (67%, compared to no more than 25% that would be expected by chance). This is consistent with previous results (Gorgoraptis et al., 2011; Pertzov & Husain, 2014), and indicates that feature binding in VWM does not entirely rely on spatial separation of stimuli even at shorter ISIs.

On the other hand, when sample items were presented at different locations, we found evidence that swap errors occurred more frequently between spatially close items, indicating a role for location in feature binding at least in the different-location condition. An increase of swap errors with spatial proximity has been observed in previous studies (Emrich & Ferber, 2012; Rerko, Oberauer, & Lin, 2014; Bays, 2016; Schneegans & Bays, 2017), but this is to our knowledge the first time this effect has been found when location was not a task-relevant feature.

Unlike Pertzov and Husain (2014), we found that in the short ISI condition, the decrease in swap errors when items were presented at different locations was largely balanced by an increase in the proportion of uniform responses, rather than an increase in the proportion of target responses. This may reflect an (intentional or implicit) strategy aimed at producing the most likely correct response from noisy memory representations, given different levels of certainty as to which memory item is being cued. This interpretation is based on evidence that the retrieved features of different sample items are associated with differing precisions, and that humans have at least partial knowledge of these precisions (Fougnie, Suchow, & Alvarez, 2012; van den Berg, Shin, Chou, George, & Ma, 2012; van den Berg, Yoo, & Ma, 2017; Schneegans, Taylor, & Bays, 2020). Consider the case that the target item in a trial is retrieved with very low precision. If the cue identifies the target item with high certainty, the participant should always attempt to produce that item’s orientation as a response, even if it is of such low precision that it is likely to be categorized as a random response in the mixture model. However, if there is uncertainty about which item is cued, it may be advantageous to report an orientation that is retrieved with high precision, even if it belongs to an item that is somewhat less likely to be the actual target. This would result in an increase of swap errors.

This account is still generally consistent with the hypothesis of Pertzov and Husain (2014) that memory for feature bindings is impaired in the same-location condition. This condition presumably led to greater uncertainty about the cued item, leading to the observed shift from uniform responses towards swap errors. However, such uncertainty does not necessarily imply an impairment of feature binding, as it would also be expected if memory for the item’s cue feature (here, color) is impaired by sequential presentation at the same location.

Critically, the effect did not generalize to the long ISI condition, where we found no positive evidence for a location effect on any parameter of the mixture model. This suggests that it is not the shared location of sample items alone that impairs recall, but the specific pairing of shared location with rapid presentation. The effect may therefore be attributed to masking or temporal crowding (Yeshurun, Rashal, & Tkacz-Domb, 2015) leading to impaired encoding of items in memory, rather than a necessary role of location for binding.

This interpretation is also consistent with the finding indicating higher swap frequencies in the short-ISI same-location condition specifically between a target and directly succeeding non-target. This effect is reminiscent of increased swap rates between directly succeeding target items reported in rapid sequential visual presentation tasks, which have likewise been explained as encoding errors (Wyble, Bowman, & Nieuwenstein, 2009; Wyble, Potter, Bowman, & Nieuwenstein, 2011). We note that the MAD measure we used to assess effects of temporal distance does not discriminate between swap errors and response biases towards non-target features. However, biases should result in decreased recall precision in the mixture model fits, which we did not observe, and therefore swap errors provide the most plausible explanation for the combined results.

We considered a possible alternative to this account, namely that the observed differences between the two ISI conditions were the result of a verbalization strategy. The longer ISI may have allowed more time for forming verbal representations that could supplement visual working memory and compensate for binding deficits in the same-location condition. Such a strategy should have resulted in more categorical responses in the long-ISI conditions. We tested this by producing scatter plots of all pairs of target feature and response feature, and density plots of responses over the space of possible orientations (Fig. S1; Hardman, 620 Vergauwe, & Ricker 2017). While we observed a strong oblique effect (Appelle, 1972; De Gardelle, Kouider, & Sackur, 2010), there were no clear signatures of responding categorically, and crucially no systematic differences in response densities between ISI conditions. This indicates that verbalization did not contribute substantially to recall performance.

## Experiment 2

Experiment 1 confirmed the key finding of (Pertzov & Husain, 2014) under the original conditions, but also found evidence that the effect does not generalize to longer ISIs. In Experiment 2, we tested whether the location effect generalizes to other feature combinations. If the increase in swap errors observed in the same-location condition at short ISI is caused by an impairment of binding memory, we should find a similar effect when memory for different visual features is tested in the same way. Here, we used color as the feature to be reported by the participant (on a continuous color wheel), and shape as the cue feature.

With this modification, we also address the possibility that orientation might represent a special case with respect to the location manipulation. First, oriented bars at different locations might be perceived as forming a single shape or be memorized as a configuration (especially if the sequential presentation is fast enough). Such configuration effects have been reported in change detection tasks for orientation stimuli (Delvenne & Bruyer, 2006).

Second, even when presented at the same location, the bars only directly overlap at their center. They could still be perceived as separate items if they were presented simultaneously, and might in fact be visualized in such an overlaid fashion. To rule out the possibility that any effects observed are specific to orientation stimuli, we opted to use colored shapes as sample stimuli, with shape as a categorical cue feature and color as continuous report feature. These stimuli are unlikely to show significant configuration effects when presented at different locations, and they overlap substantially when presented at the same location. Even though the stimuli we used do not cover exactly the same area, we consider it very unlikely that participants could have distinctly perceived and memorized the small non-overlapping regions of the shapes at the eccentricity at which they were presented (Burkhalter & Van Essen, 1986; Poder & Wagemans, 2007).

### Methods

**Fig. 6:**
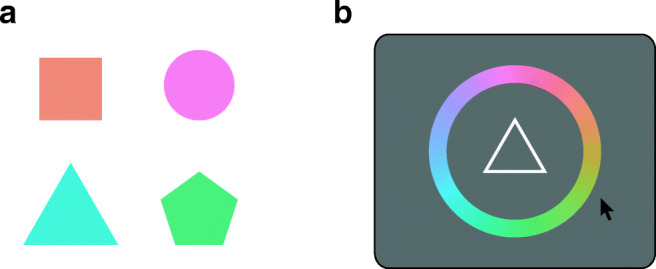
Examples of stimuli (**a**) and a response display with shape cue and color wheel (**b**), as used in Experiment 2

Twenty different participants (14 female, mean age 22.2 years), all with normal or corrected-to-normal visual acuity and normal color vision, performed Experiment 2 after giving informed consent. Apparatus, procedure, and conditions were identical to Experiment 1, but the sample stimuli were colored shapes (Fig. 6). The four shapes presented in each trial were: circle, equilateral triangle, square, and equilateral pentagon (in random order), with the surface area of each shape normalized to 2 dva^2^. When presented at the same location, the overlap in area for any two shapes was at least 80%, and no point in any shape was more than 0.5 dva removed from the closest point in any other shape.

The colors were chosen from a circle in CIELAB color space with a fixed luminance *L* = 74, centered in the *ab*-plane at (0,0) and with a radius of 40. Color values are given as angles on this color wheel in degree, covering the range [0^∘^,360^∘^). For each item, a color angle was drawn from a circular uniform distribution, with the constraint that the color angle between any two items in each trial differed by at least 20^∘^. Placement and timing of the sample stimuli were the same as in Experiment 1.

Following the memory delay, the central fixation point was replaced by a cue in the form of a white outline of one of the shapes. Once the participant moved the mouse, a color wheel appeared around this shape, and the shape cue was filled with a probe color when the mouse pointer was moved over the color wheel. The participant made a response by clicking on the color wheel.

We applied the same mixture model fits and Bayesian statistical analyses as in Experiment 1, and also applied the same Bayesian stopping criterion. The only difference is that here, the circular response feature space (color hue) covers 360^∘^, instead of 180^∘^ for orientation. Consequently, the heuristic measures for the proportion of target responses and swap errors include all responses within ± 30^∘^ from the target or non-target feature values, respectively.

### Results

In Experiment 2, participants reported the color of one of four sequentially presented shapes based on a shape cue, in a blocked design with two location conditions (same or different) and two ISI durations (300 or 600 ms).

**Fig. 7:**
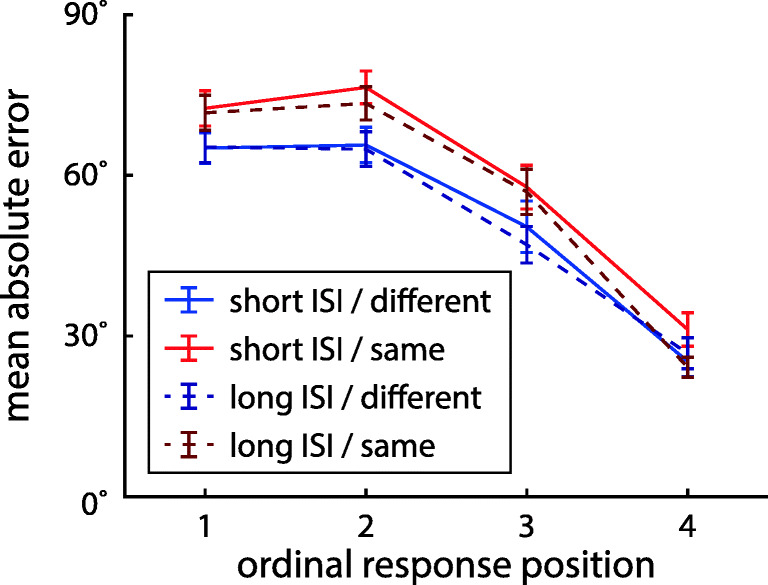
Mean absolute error in color response (as angle on a color wheel) for target items at different ordinal positions in each task condition of Experiment 2. Error bars indicate ± 1 SE

We again performed a three-factor Bayesian ANOVA for the effects of location, ISI, and ordinal position of the cued item on mean absolute response errors (Fig. 7). As in Experiment 1, we found overwhelming evidence for an effect of ordinal position (BF_incl_ = 2.44 ⋅ 10^67^), but now we also found very strong evidence for an effect of the location condition (BF_incl_ = 3411), with higher recall performance if stimuli were presented at different locations. For the ISI condition and all interactions, the ANOVA produced moderate to strong evidence against an effect (all BF_incl_ between 0.04 and 0.25).

**Fig. 8:**
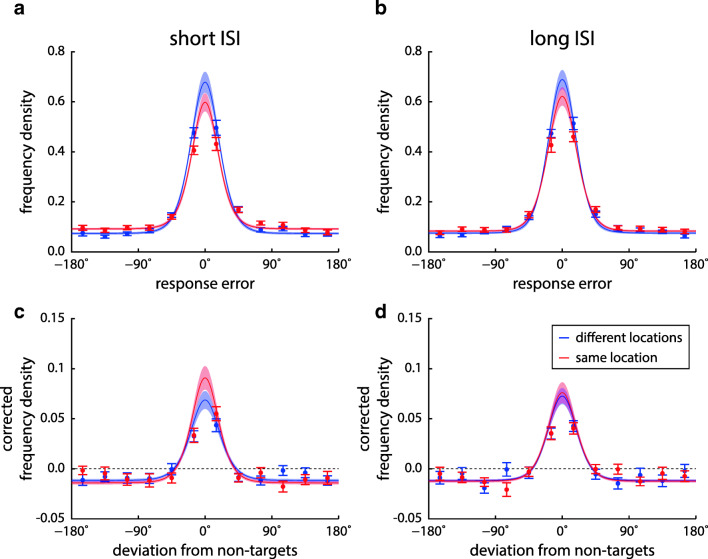
Response distributions relative to target (**a** & **b**) and non-target feature values (**c** & **d**) for different experimental conditions in Experiment 2, and corresponding mixture model fits. Histograms are shown in the same format as in Fig. 3

**Fig. 9:**
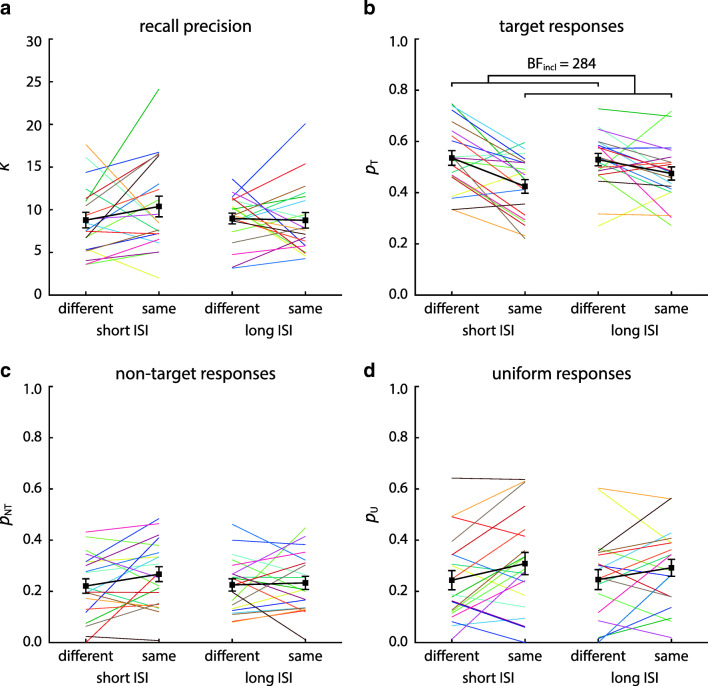
Parameter estimates of mixture model fits for Experiment 2. Results are shown in the same format as in Fig. 4, but note that the scale for concentration parameters is changed due to substantially higher recall precision in this task. The Bayes factor in (**b**) is the evidence in favor of including a main effect of location

We applied the mixture model to each participant’s data to distinguish different kinds of error. The response distributions relative to the target value and the non-target values for each condition as well as the corresponding model fits are shown in Fig. 8, and estimated parameters of the mixture model are shown in Fig. 9.

The Bayesian stopping criterion was not reached in this experiment within the predefined limit of 20 participants, reaching only weak evidence against an interaction of location and ISI conditions on the proportion of swap errors (expressed as difference of differences, BF_10_ = 0.37; Fig. 9c). A subsequently performed Bayesian ANOVA likewise showed weak evidence against an interaction effect (BF_incl_ = 0.46), as well as weak to moderate evidence against an effect of location (BF_incl_ = 0.56) and ISI (BF_incl_ = 0.30).

However, we obtained different results when we used the heuristic measure of swap errors, namely the proportion of responses within ± 30^∘^ from non-target features (two central bins of the histogram in Fig. 8c and d). Here, a Bayesian ANOVA yielded weak evidence against an effect of ISI (BF_incl_ = 0.42), but moderate evidence in favor of an effect of location (BF_incl_ = 5.89). For the interaction, the results were inconclusive (BF_incl_ = 1.21). Subsequent Bayesian *t* tests showed that the location effect was driven by the short ISI condition (BF_10_ = 9.84), with a higher proportion of swap errors when stimuli were presented at the same location. For the proportion of swap errors in the long ISI condition, we found moderate evidence against a modulation by location condition (BF_10_ = 0.28). These results are more in line with the findings from Experiment 1.

We again applied the analyses also to the other parameters of the mixture model. The concentration parameter *κ* (Fig. 9a) showed no modulation by the experimental conditions, with weak to moderate evidence against an effect of location (BF_incl_ = 0.32), ISI (BF_incl_ = 0.33) and an interaction (BF_incl_ = 0.51). In contrast, we found very strong evidence for an effect of location condition on the proportion of target responses (BF_incl_ = 284; Fig. 9b), with more target responses in the different-location condition than in the same-location condition. The ANOVA further produced weak evidence against an effect of ISI (BF_incl_ = 0.40) or an interaction (BF_incl_ = 0.73). These results were confirmed by the heuristic measure of target responses (two central bins in the histograms in Fig. 8), producing even stronger evidence for an effect of location (BF_incl_ = 3593) and weak evidence against effects of ISI (BF_incl_ = 0.53) and an interaction (BF_incl_ = 0.38). Finally, for the proportion of uniform responses (Fig. 9d), we found only weak evidence for an effect of location (BF_incl_ = 1.46), and moderate evidence against an effect of ISI (BF_incl_ = 0.23) or an interaction (BF_incl_ = 0.32).

**Fig. 10:**
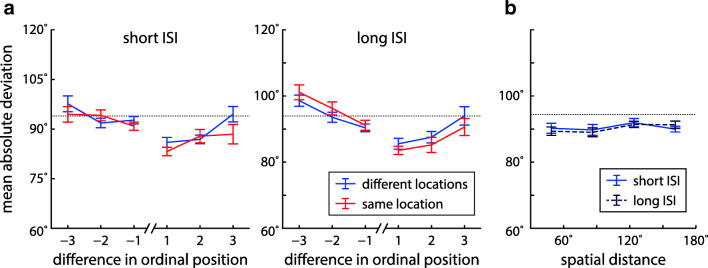
Effects of temporal and spatial proximity of non-targets on color responses in Experiment 2, displayed in the same format as in Fig. 5

We also assessed the effects of temporal and spatial distance between target and non-target items on the occurrence of swap errors, in the same way as for Experiment 1 (Fig. 10). A three-factor ANOVA showed strong evidence for an effect of temporal separation (BF_incl_ = 4.13 ⋅ 10^21^), with MADs below chance level for non-target items in the two ordinal positions following the target (Table S3). There was moderate to strong evidence against effects of location (BF_incl_ = 0.25), ISI (BF_incl_ = 0.16), and all interactions (all BF_incl_ between 0.05 and 0.21). We found no modulation of MADs by spatial distance in this experiment (Fig. 10b; moderate evidence against an effect of spatial distance, BF_incl_ = 0.19, ISI, BF_incl_ = 0.18, and interaction, BF_incl_ = 0.10). The fact that MADs for all location bins were lower than the expected value (Table S4) is likely merely due to swap errors based on temporal proximity, which could occur at all spatial distances.

### Discussion

In this experiment, with shape as cue feature and color as reported feature, we did find a consistent effect of the location condition on recall errors, without a modulation by ISI duration. However, the effect is different from the one observed in the previous experiment: We found a decrease in the proportion of target responses when items were presented at the same location (which is consistent with the original findings of Pertzov and Husain 2014), but no consistent increase in the proportion of swap errors. Our results for the proportion of swap errors in the short ISI condition were somewhat ambiguous. We did not find any effects of stimulus location based on the mixture model parameters (which we consider to be the more reliable measure), but there was evidence for such an effect using the heuristic method. Critically however, the effect did not extend to the long ISI condition in either of the two approaches.

One caveat with respect to the comparison of results across the two experiments is the difference in sample size (due to the Bayesian stopping criterion being reached in Experiment 1, but not Experiment 2). It is possible that we would have found an effect on the proportion of target responses also in Experiment 1 if we had collected more data. However, the stopping criterion should not have biased the results regarding the proportion of swap errors as the main variable of interest (Rouder, 2014).

It should be noted that the decrease in the proportion of target responses which we consistently observed must necessarily be accompanied by an increase in the proportion of uniform responses or swap errors (or both), as these proportions must sum to one. We did observe a numerical increase in both of these measures (Fig. 9), and the lack of evidence for any consistent effects suggests that the cause for the decrease in target responses varies across participants.

Thus, we cannot rule out that the decrease in target responses is still caused by an impairment in feature binding. The analysis of temporal separation effects for this experiment suggests that responses tended to deviate towards the features of non-targets that succeeded the target item, compared to more symmetrical effects for directly preceding and succeeding non-targets observed in Experiment 1 (see Tables S1 and S3). It is possible that color stimuli are susceptible to overwriting, rather than confusion of the memorized features of multiple object. Overwriting could result either in swap errors (e.g., if a participant is not aware of this) or in uniform responses for a cued feature.

Such an effect is also compatible with the results of Experiment 1, where color is used as cue feature. If memory for color is impaired but the orientations can still be remembered reliably (e.g., because they can be visualized as an overlaid pattern, as suggested above), then a selective increase in swap errors would be expected. The differences in the time structure of the observed effects—with impairment only at short ISIs in Experiment 1, but extending to long ISIs in Experiment 2—may be due different demands on color memory in the two tasks. In Experiment 1, the same four categorical color values were used in all trials, whereas in Experiment 2, four novel colors had to be memorized in each trial and later reported on a continuous scale. The latter may have required more time for encoding the color values, and consequently have led to greater interference between sample stimuli even at longer ISIs.

Although we used continuous colors as report features in this experiment, it is still possible that participants generated verbal labels to support their memory. To assess the contribution of verbal (and therefore categorical) memory to task performance, we again generated scatter plots and response density plots over the response feature space (Fig. S2). As in Experiment 1, we did observe some biases in the response behavior, but no strong clustering in responses and no systematic difference in response densities between short and long ISI condition. We therefore believe that verbal or categorical memory did not contribute substantially to the response performance in this task.

## General discussion

The present study successfully replicated the key finding of Pertzov and Husain (2014) when we used the same stimulus settings: The proportion of swap errors in orientation recall was selectively increased when sample items were presented sequentially at the same location rather than at different locations. Critically, however, this result did not generalize to a task condition with a longer time interval between sample items, where we found no significant effects of location condition. In addition, the finding did not generalize to different feature values. In a color report task, we did find decreased recall performance overall when sample stimuli were presented at the same location, but this performance deficit could not be attributed to a specific increase in swap errors.

These results point to encoding interference between sample stimuli as the cause of impairment (Yeshurun et al., 2015; Wyble et al., 2011), rather than an inability to maintain feature bindings when items are presented at the same location. This resolves an apparent conflict between the results of Pertzov and Husain (2014) and those of Harrison and Bays (2018), who did not find any impairment in recall performance when sample items were presented in close proximity to each other. The different outcomes can now be explained as an effect of the differences in stimulus timing, and the observed time scales of interference effects are consistent with previous studies (Ahmad et al., 2017; Ricker & Hardman, 2017).

What do these results mean for our understanding of visual feature binding? One possible interpretation would be that space does not take a special role in individuating objects and maintaining the binding between other visual features. However, it must be noted that the present study only compared performance for different forms of sequential presentation of sample items. Our observations are not in direct conflict with various studies that support a privileged role of space when sample arrays are presented simultaneously (Treisman & Zhang, 2006; Schneegans & Bays, 2017; Rajsic et al., 2017; Kovacs & Harris, 2019). They also do not contradict previous findings that memory performance is impaired with sequential compared to simultaneous presentation (Gorgoraptis et al., 2011; Allen et al., 2006; Allen, Baddeley, & Hitch, 2014).

A more parsimonious interpretation of our results is that the role of item location is reduced or nullified specifically when items are presented sequentially (with sufficient delays to avoid interference). This may be due to a lack of directly available configuration (Jiang, Olson, & Chun, 2000) or relational spatial information (Hollingworth, 2007), both of which have been found to play an important role in VWM. It is therefore possible that in sequential presentation paradigms, features like color and orientation are bound directly to each other, in line with classical object-based accounts of memory storage (Luck & Vogel, 1997; Luria & Vogel, 2011).

An alternative explanation is that time may take over the role of space in mediating feature binding when sample stimuli are presented sequentially. If each individual feature in working memory is associated with the time at which it was encoded (either as a continuous variable or as an ordinal position), then the features belonging to the same object may be identified by their matching timing information. This is directly analogous to the proposal that features in separate feature maps are bound to each other only via their shared location (Schneegans & Bays, 2017).

The role of time in feature binding has received less attention than that of space in the VWM literature (Manohar, Pertzov, & Husain, 2017; Schneegans & Bays, 2019). However, several studies have found that performance in recalling the sequential order of visually presented objects is comparable to performance for location recall (van Asselen et al., 2006; Delogu, Postma, & Nijboer, 2012; Rondina, Curtiss, Meltzer, Barense, & Ryan, 2017), and have variously argued that either time or space takes a dominant role in structuring working memory representations. Furthermore, a recent study found evidence that presentation time, like stimulus location, is incidentally encoded in VWM even when it is not task-relevant (Heuer & Rolfs, 2020).

Analogous roles of time and space are also supported by the present finding that swap errors tended to occur predominantly between successive items in the presentation sequence, which matches previous results (Sapkota, Pardhan, & van der Linde, 2016) and is analogous to the greater proportion of swap errors observed between spatially proximal items (Emrich & Ferber, 2012; Rerko et al., 2014; Bays, 2016; Schneegans & Bays, 2017). To date, however, no study has explicitly investigated the role of time in mediating binding between multiple visual surface features in working memory.

Some computational models have addressed the role of time in feature binding. The interference model of Oberauer and colleagues describes memorization in working memory as formation of associations between a context dimension and a feature to be reported, with the context dimension typically being ordinal position in the case of verbal working memory (Oberauer, Lewandowsky, Farrell, Jarrold, & Greaves, 2012), and spatial location for VWM (Oberauer & Lin, 2017). In principle, time or ordinal position may also be used as context for visual features, but the model does not specify what the relation between these different context dimensions would be.

The binding pool model (Swan & Wyble, 2014) considers temporal order as the dominant feature dimension that mediates binding between other visual features. Earlier versions of the same model were used to explain attentional blink and swap errors between successive items in rapid serial visual presentation tasks (Bowman & Wyble, 2007; Wyble et al., 2009; Wyble et al., 2011). These errors arise in the model due to binding of multiple objects to the same ordinal position. For simultaneously presented stimulus arrays, the model assumes that items are attended sequentially, imposing a temporal order that mediates binding between other features, including object locations.

A complementary approach is taken by the population coding model of Schneegans and Bays (2017), which proposes that different visual features are encoding by separate feature maps over space, and bound to each other only by their shared location. This mechanism of binding via space has successfully accounted for error correlations in double-report experiments (Schneegans & Bays, 2017; Kovacs & Harris, 2019). It has been suggested that such an architecture could deal with stimuli presented sequentially at the same location by internally remapping them to different locations—for instance, along a horizontal axis to preserve order information (Abrahamse, Van Dijck, Majerus, & Fias, 2014). However, to date no experimental evidence for such a process has been found.

Alternatively, neural populations underlying VWM may show sensitivity to both location and presentation time, either in a mixed code (with different subsets of neurons sensitive to either location or time, in addition to visual features) or in a fully conjunctive code. Additional studies that manipulate both presentation time and location will be necessary to elucidate the underlying memory mechanisms.

### Conclusions

In this study, we found that presenting sample items sequentially at the same location does not necessarily impair memory for feature bindings, as long as sufficient time is given for encoding each item. This shows that binding between visual features does not only rely on a spatial separation between different objects, and suggests that presentation time can likewise serve to individuate objects in VWM. How exactly time or sequential order is represented in VWM, and how it interacts with space, represent important questions for future research.

## Electronic supplementary material

Below is the link to the electronic supplementary material.

(PDF 9.59 MB)

## Data Availability

Data and analysis code associated with this study is publicly available at https://osf.io/vcq5n/.
