# Supplementary material for: Location-independent feature binding in visual working memory for sequentially presented objects
Source: Atten Percept Psychophys. 2021 Apr 16;83(6):2377–93. doi: 10.3758/s13414-021-02245-w (PMC8302549; doi:10.3758/s13414-021-02245-w)
Supplement: Supplementary file 1 — (PDF 9.59 MB) [file 13414_2021_2245_MOESM1_ESM.pdf]

Supplementary Information

Location-independent feature binding in visual working memory for sequentially presented objects

Sebastian Schneegans, William J. Harrison, Paul M. Bays
Department of Psychology, University of Cambridge, Cambridge, UK

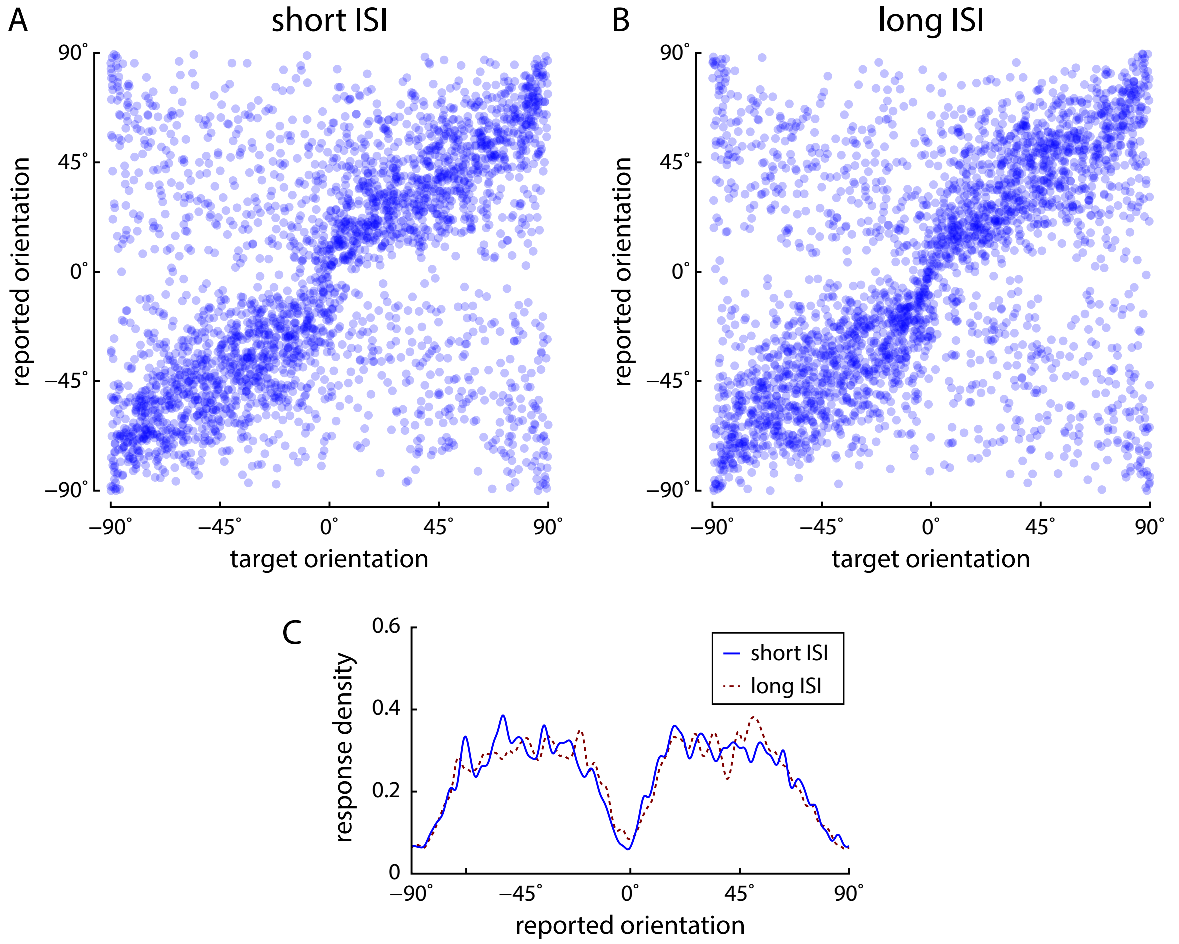

Figure S1: Scatter plots and response densities in Experiment 1. (A) Response feature value plotted against target feature value for each trial of the short-ISI conditions, pooled across location conditions and participants. Perfect performance would produce points aligned on the main diagonal, while purely categorical response behavior should produce horizontal streaks in the scatter plot (Hardman et al., 2017). We do observe response biases away from the cardinal directions, consistent with an oblique effect (Appelle, 1972), but no clustering of response values. (B) The same plot for the long-ISI conditions. (C) Density of response values over the space of possible orientations. The distribution was generated by convolving a delta function for each individual response with a sharp von Mises distribution ($\kappa = 400$, scaled down to the range $[-90°, 90°)$).

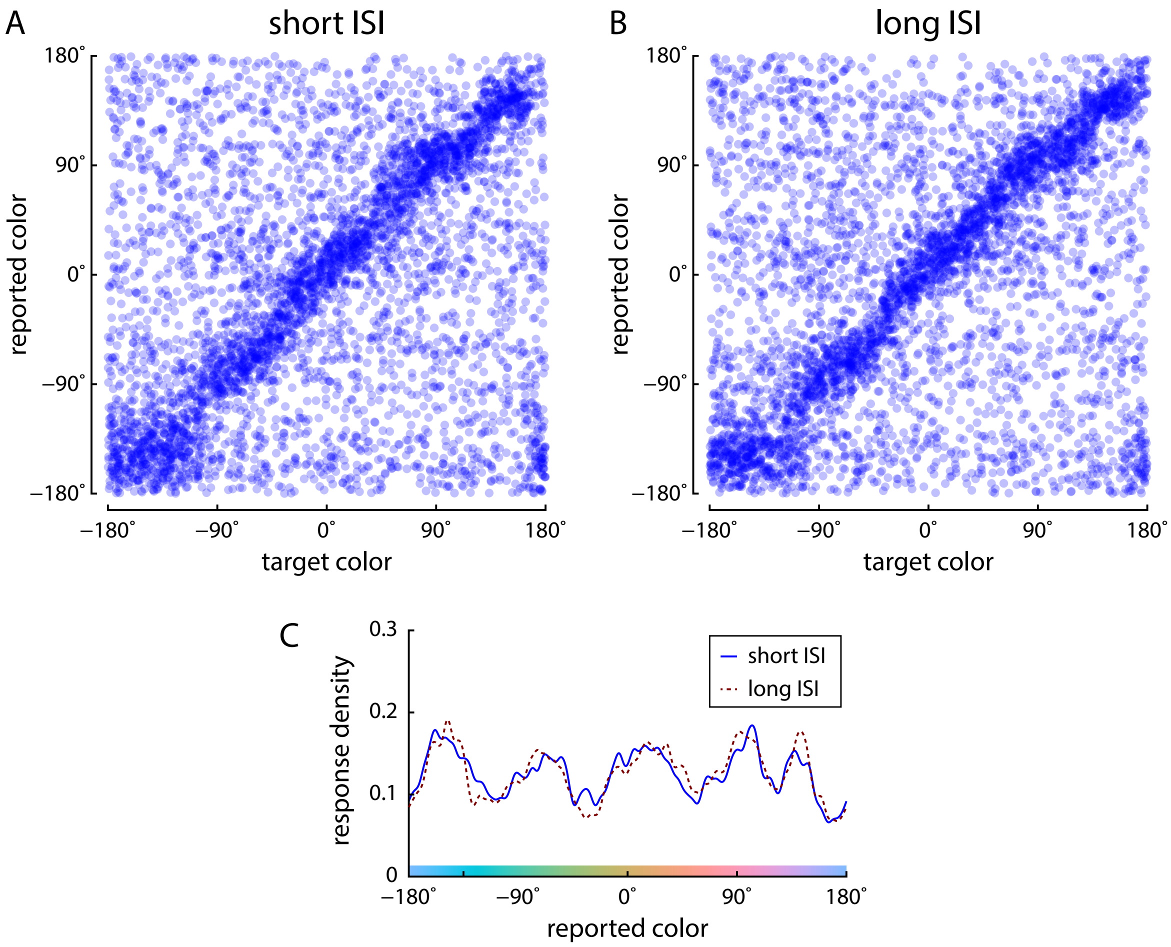

Figure S2: Scatter plots and response densities in Experiment 2, in the same format as in Figure S1.

Table S1: Evidence for swap errors at different temporal separations between target and non-target items in Experiment 1. For each condition and relative ordinal position of the non-target items relative to the target, we determined the mean absolute deviation (MAD) of the response from the non-target feature values (see Fig. 5A). We then compared these values to the values expected in the absence of swap errors (determined by shuffling non-target features across trials, see Methods: Response distributions and mixture model fits). We performed paired-sample Bayesian t-tests, and report the evidence in favor of a difference, BF_{10}, without correction for multiple comparisons. Results for comparisons in which the MAD was higher than the value expected in the absence of swap errors are shown in parentheses. Results for comparisons in which the MAD was lower than the expected value (thus indicating the occurrence of swap errors), and in which we found at least moderate evidence for a difference ($BF_{10} > 3$), are printed in bold.

	difference in ordinal position					
**condition**	-3	-2	-1	1	2	3
short / different	(4.96)	0.311	0.600	**3.98**	2.70	(1.47)
short / same	0.337	0.412	**12.1**	**5644**	(0.419)	(1.74)
long / different	(0.298)	(0.316)	**7.28**	**4.08**	1.04	(7.55)
long / same	(1.31)	0.289	**5.15**	**34.8**	0.305	(1.39)

Table S2: Evidence for swap errors at different spatial distances between target and non-target items in Experiment 1. Bayes factors were determined and are shown in the same form as in Table S1, but now for different angular distance bins (as in Fig. 5B).

	spatial (angular) distance			
**condition**	$[0°, 67.5°)$	$[67.5°, 105°)$	$[105°, 142.5°)$	$[142.5°, 180°]$
short / different	0.669	**95.1**	(0.302)	0.288
long / different	**62.7**	2.27	0.594	(0.407)

Table S3: Evidence for swap errors at different temporal separations between target and non-target items in Experiment 2 (Fig. 10A), in the same format as in Table S1.

	difference in ordinal position					
**condition**	-3	-2	-1	1	2	3
short / different	(0.522)	1.67	1.50	**1755**	**1138**	0.233
short / same	(0.269)	(0.269)	0.818	$\mathbf{2.78 \cdot 10^5}$	**3.00**	0.768
long / different	(5.26)	0.256	**9.07**	**400**	**44.2**	0.233
long / same	(12.1)	(0.456)	0.94	$\mathbf{7.38 \cdot 10^4}$	**36.6**	0.454

Table S4: Evidence for swap errors at different spatial distances between target and non-target items in Experiment 2 (Fig. 10B), in the same format as in Table S1.

	spatial (angular) distance			
**condition**	$[0°, 67.5°)$	$[67.5°, 105°)$	$[105°, 142.5°)$	$[142.5°, 180°]$
short / different	**8.08**	**15.0**	1.73	**2672**
long / different	**47.0**	**49.6**	**7.59**	**4.05**

References

Appelle S (1972) Perception and discrimination as a function of stimulus orientation: the" oblique effect" in man and animals. Psychological bulletin 78(4):266

Hardman KO, Vergauwe E, Ricker TJ (2017) Categorical working memory representations are used in delayed estimation of continuous colors. Journal of Experimental Psychology: Human Perception and Performance 43(1):30
